# Perinatal care during the COVID-19 pandemic in the state of Paraná

**DOI:** 10.1590/0034-7167-2025-0205

**Published:** 2026-03-30

**Authors:** Emily Marques Alves, Adriana Zilly, Lhays Emilly da Silva Moraes Ribeiro, Keli Regiane Tomeleri da Fonseca Pinto, Catia Campaner Ferrari Bernardy

**Affiliations:** I Universidade Estadual de Londrina. Londrina, Paraná, Brazil; II Universidade Estadual do Oeste do Paraná. Foz do Iguaçu, Paraná, Brazil

**Keywords:** Perinatal Care, Apgar Score, COVID-19, Pregnancy, Patient-Centered Care., Atención Perinatal, Puntaje de Apgar, COVID-19, Embarazo, Atención Centrada en la Persona.

## Abstract

**Objectives:**

to assess the association between good obstetric practices in the perinatal period and newborn vitality measured by Apgar during the COVID-19 pandemic in Paraná.

**Methods:**

a cross-sectional study, excerpt from the research “Coping with COVID-19 and maternal and child care”, with postpartum women treated in hospitals in five Regional Health Districts of Paraná during the COVID-19 pandemic (2021 and 2022). Statistical analysis used frequencies, means, and standard deviations. Association between Apgar and good obstetric practices was analyzed using chi-square and Fisher’s exact tests.

**Results:**

a total of 1,503 women were included. The variables referral hospital, skin-to-skin contact, and presence of a companion had positive outcomes on Apgar scores.

**Conclusions:**

despite the adversities of the pandemic, some good practices were maintained, and Apgar score was positive. However, the continued need for coordination between managers and professionals, and public policies to ensure perinatal care quality in the state is highlighted.

## RESUMEN

**Objetivos:**

evaluar la asociación entre las buenas prácticas obstétricas en el período perinatal y la vitalidad neonatal medida mediante el Apgar durante la pandemia de COVID-19 en Paraná.

**Métodos:**

estudio transversal, extracto de la investigación “Afrontamiento de la COVID-19 y atención maternoinfantil”, con puérperas atendidas en hospitales de cinco Distritos Regionales de Salud de Paraná durante la pandemia de COVID-19 (2021 y 2022). El análisis estadístico se realizó mediante frecuencias, medias y desviaciones estándar. La asociación entre el Apgar y las buenas prácticas obstétricas se analizó mediante las pruebas de chi-cuadrado y exacta de Fisher.

**Resultados:**

se incluyeron 1.503 mujeres. Las variables hospital de referencia, contacto piel con piel y presencia de acompañante mostraron resultados positivos en la puntuación de Apgar.

**Conclusiones:**

a pesar de las adversidades de la pandemia, se mantuvieron algunas buenas prácticas y la puntuación de Apgar fue positiva. Sin embargo, se destaca la necesidad de coordinación entre gestores, profesionales y políticas públicas para garantizar la calidad de la atención perinatal en el estado.

## INTRODUCTION

The process of pregnancy and childbirth is physiological, familial, and social. However, with modernization and industrialization, the proposed care model has become overly medicalized. The movement to restore humanized care has been a topic of discussion both globally and nationally, but it still faces challenges, one of which has been the COVID-19 pandemic. Changes in healthcare and restrictions on social interaction during the pandemic have intensified negative feelings among pregnant women, increasing the risk of emotional instability and, consequently, maternal-fetal complications^(1,2)^.

In 2021, the World Health Organization (WHO) analyzed 135 countries and found that 54% changed prenatal care and 31% changed childbirth services, contributing to the increase in stillbirths and maternal deaths^(3,4)^. The pandemic has further highlighted the fragility of global obstetric care, weakening the link between women and healthcare services^(5)^.

For over two decades, the WHO has recommended guidelines for healthy care for both mothers and babies. In 2018, it reinforced and updated best practices for childbirth through the “Intrapartum Care for a Positive Childbirth Experience” manual. Based on human rights, the manual proposes a model of intrapartum care centered on women’s and newborns’ needs, aiming to reduce maternal and neonatal morbidity and mortality rates and thus strengthen safe and positive experiences for women and their families^(6)^.

During the pandemic, there was a setback in WHO recommendations for the five stages of intrapartum care as well as prenatal care. Uncertainties surrounding COVID-19 led healthcare services to adopt restrictions and interventions without scientific support^(7)^. Qualitative studies carried out in various regions of Brazil indicate that some measures such as the restriction of companions during labor, childbirth, and postpartum, criteria for skin-to-skin contact, early umbilical cord clamping, start of telehealth, cancellation or postponement of consultations and tests, and non-attachment of women to the reference maternity hospital were seen as necessary to maintain the safety of patients and professionals^(7-10)^.

In Turkey, during the COVID-19 pandemic, pregnant women sought obstetric emergency care more frequently due to prenatal care interruption. As a result, there was an increase in cases of small-for-gestational-age babies, Apgar scores <7 at 5 minutes, and hypoxic-ischemic encephalopathies^(11)^. In a meta-analysis, the increase in prematurity was reported as an event associated with the pandemic^(12)^.

The mother and child dyad represents a unique population in healthcare, so it is essential to reinforce the use of good obstetric practices achieved over the years^(13)^. Understanding how adaptations made to obstetric care during the COVID-19 pandemic impacted fetal outcomes is an important source of information for organizing future work processes, in addition to contributing to Goal 3 (Quality Health) of the Sustainable Development Goals.

Therefore, given all of the above and the relevance of this topic, the question is: were good practices in care for pregnant women implemented during the COVID-19 pandemic in the state of Paraná? How did the care provided to pregnant women during this period impact fetal well-being?

## OBJECTIVES

To assess the association between good obstetric practices in the perinatal period and newborn vitality measured by Apgar during the COVID-19 pandemic in Paraná.

## METHODS

### Ethical aspects

This study followed national and international ethical guidelines, and was approved by the Research Ethics Committees of all participating universities. All participants were briefed on the research, and interviews began only after they signed the Informed Consent Form.

### Study design, period and location

This is a cross-sectional study, part of multicenter research entitled “Coping with COVID-19 and maternal and child care” (In Portuguese, *Enfrentamento da COVID-19 e a assistência materno-infantil*), which analyzed perinatal care and child follow-up during the COVID-19 pandemic between 2021 and 2022. This study was conducted at lowand high-risk maternity hospitals in municipalities belonging to five Health Regions (HRs) of the state of Paraná: the 3^rd^ HR, located in the east; the 9^th^ and 10^th^ HRs, located in the west; the 15^th^ HR, located in the northwest; and the 17^th^ HR, located in the north of the state. The methodology followed all STrengthening the Reporting of OBservational studies in Epidemiology recommendations.

### Population or sample; inclusion and exclusion criteria

Sample calculation was based on the number of births in 2020 in each HR, considering a 5%margin of error, with a 95% confidence level and an additional 10% safety margin, due to the characteristics of the original research, as it was a follow-up study and losses could occur during the period, resulting in the calculation of 2,112 women.

This study used data only from the first phase of the original research, which involved in-person interviews in maternity wards with 1,830 women: 432 from the 3^rd^ HR, 408 from the 9^th^ HR, 418 from the 10^th^ HR, 255 from the 15^th^ HR, and 317 from the 17^th^ HR. Postpartum women who gave birth during the COVID-19 pandemic and resided in the municipalities of the aforementioned HRs were included. Women with any condition that would interfere with the progress of this research were excluded. For analysis purposes, participants with two or more variables missing from the database were excluded. To assess sample sufficiency after losses, a post-hoc power analysis was performed for chi-square test (χ^2^) using G*Power software (version 3.1), considering chi-square test with 5 degrees of freedom, a significance level of 5% (α = 0.05), and determining the effect size considered large (w = 0.5), according to Cohen’s criteria. The result indicated a statistical power of 100% (1 - β = 1.0), demonstrating that the effective sample was adequate to detect associations with high magnitude between the analyzed variables^(14)^.

### Study protocol

The original data collection instrument consisted of 112 questions; of these, 20 were analyzed. Independent variables were related to sociodemographic data such as age, self-reported skin color, marital status, education level, paid employment, family income, and receipt of emergency aid. Other independent variables comprised the set of variables defined as good obstetric practices, such as prenatal care (more than six consultations and gestational age less than 14 weeks at the first consultation), referral to a referral hospital, risk stratification, spontaneous membrane rupture, non-pharmacological pain relief, presence of a companion, skin-to-skin contact, and delayed umbilical cord clamping. The outcome variable was Apgar scores at 1 and 5 minutes.

The Apgar score is an immediate assessment of neonatal condition, used as an indicator of mortality and an effective way to compare the results of obstetric practices. The test consists of five variables: heart rate; respiratory effort; muscle tone; reflex response; and skin color. These variables are each scored between 0 and 2 and are initially assessed at the first and fifth minutes of a newborn’s life^(15,16)^.

Apgar scores of 7 and above are considered reassuring, representing good fetal health. Scores below this level are a warning sign, as they are associated with a higher risk of perinatal asphyxia and cerebral palsy, leading to increased newborn mortality in the first week of life^(15-17)^. Therefore, the Apgar score is presented in this research, dichotomized into two categories: ≤ 6 and ≥7 points.

### Analysis of results and statistics

Data were analyzed using descriptive and inferential statistics using IBM SPSS Statistics version 29.0. Categorical variables were presented as absolute (n) and relative (%) frequencies. Quantitative variables, both discrete and continuous, were expressed as means and standard deviations (SD).

Initially, the analysis was conducted considering the total number of women in the sample, followed by stratification by mode of birth. To analyze the hypothesis of an association between newborn vitality measured by Apgar and independent variables of good obstetric practices, Pearson’s chi-square test was applied. When the expected frequencies in the contingency table were less than 5, Fisher’s exact test was used.

## RESULTS

A total of 1,503 participants were eligible, with 327 being excluded due to missing data for analysis. The predominant self-reported skin color was white (863; 57.4%), followed by brown (479; 32.0%). Most of them (1,315; 87.5%) had a partner and had experienced one to two previous pregnancies (733; 48.8%). Regarding education, only 509 women (33.9%) had more than 13 years of schooling. The mean age was 26.61 years (SD ± 6.01), with the youngest being 13 and the oldest, 45 years. Concerning occupation, 767 women (51%) did not have paid work, and the mean family income was one to two minimum wages for 1,006 women (66.9%) (minimum wage in effect in 2021: R$ 1,100.00). A large portion of them (1,131; 75.2%), did not receive emergency aid.

Prenatal care was attended by 1,480 women (98.5%), with the majority (1,379; 91.7%) linked to the referral hospital. The women underwent risk stratification upon arrival at the hospital, with 89.94% classified as low risk. Regarding gestational age at birth, 1,368 (91.01%) were over 37 weeks gestation. As for membrane rupture, it was identified that the majority was spontaneous, with a prevalence in vaginal delivery (Table 1).

**Table 1:** Care and obstetric variables of postpartum women who gave birth in maternity hospitals of the Paraná Regional Health Districts during the COVID-19 pandemic, Paraná, 2021-2022

Variables	Vaginal delivery (n and %)	Caesarean section (n and %)
**Prenatal care** Yes No	71298.88 81.12	76898.08 151.92
**Reference hospital** Yes No No registration	64890.0 719.86 10.14	73193.35 516.51 10.14
**Risk stratification** Regular Intermediate High No record	39254.44 517.08 8611.94 19126.54	27835.50 759.57 23636.14 19418.79
**Gestational age** Moderately preterm (31-35 s) Borderline preterm (36 s) Full-term (≥37 s) No record	50.69 141.96 64990.13 527.22	141.78 121.53 71991.82 384.87
**Spontaneous rupture of membranes** Yes No	59782.9 12317.1	52967.6 25432.4
**Non-pharmacological method** Yes No	55276.7 16823.3	30038.3 48361.7
**Non-pharmacological method type** Relaxing bath Massage Using the ball Other methods	48767.6 27137.6 30642.5 8912.5	14318.3 7910.1 8611.0 141.8
**Presence of companion** Yes No	64289.2 7810.8	66985.4 11414.6
**Skin-to-skin contact** Yes No	52272.5 19827.5	38449.0 39951.0
**Timely cord clamping** Yes No	24233.6 47866.4	8110.3 70289.7

The use of non-pharmacological methods for pain relief was more frequent in women who progressed to vaginal delivery. Relaxing baths were the most frequently used practice in both outcomes (67.6% and 18.3%, respectively). A companion was present in 1,311 cases (87.2%). Skin-to-skin contact immediately after childbirth was provided to 906 women (60.3%), being more frequent in vaginal deliveries. Timely cord clamping was present in only 323 cases (21.5%), which highlights the low use of this practice (Table 1). Cesarean section was the most frequent mode of childbirth (783; 52.1%).

When analyzing the association between Apgar and all variables named as good obstetric practices in general analysis, 1^st^-minute Apgar was considered associated with referral to a reference hospital (p=0.022), and 1^st^-minute and 5^th^-minute Apgar, with skin-to-skin contact (p=0.048 and p=0.028) (Table 2).

**Table 2:** Relationship between good obstetric practices and Apgar scores at 1 and 5 minutes of newborn life (general analysis), Paraná, 2021-2022

	1^st^-minute Apgar		*p* value	5^th^-minute Apgar		*p* value
	n ≤ 6 (%)	n ≥7 (%)		n ≤ 6 (%)	n ≥7 (%)	
Prenatal Yes No	8 0.5% 0 -	1472 99.5% 23 100.0%	0.884^*^	11 0.7% 0 -	1469 99.3% 2 100.0%	0.843^*^
**Reference hospital** Yes No	5 0.4% 3 2.5%	1374 99.6% 119 97.5%	0.022^*^	8 0.6% 3 2.5%	1371 99.4% 119 97.5%	0.053^*^
**Risk stratification** Low Intermediate High No record	4 0.6% 0 - 3 0.9% 1 0.3%	666 99.4% 126 100.0% 319 99.1% 384 99.7%	0.525#	5 0.7% 1 0.8% 4 1.2% 1 0.3%	665 99.3% 125 99.2% 318 98.8% 384 99.7%	0.504#
**Gestational age** Moderately premature Borderline premature Full term No record	0 - 0 - 7 0.5% 1 1.1%	19 100% 26 100% 1361 95.5% 89 98.9%	0.844#	0 - 1 3.8% 9 0.7% 1 1.1%	19 100% 25 96.2% 1359 99.3% 1492 98.9%	0.273#
**Membrane rupture** Yes No	6 0.5% 2 0.5%	1120 99.5% 375 99.5%	0.677^*^	9 0.8% 2 0.5%	1117 99.2% 375 99.5%	0.452^*^
**Non-pharmacological method** Yes No	5 0.6% 3 0.5%	847 99.4% 648 99.5%	0.516^*^	7 0.8% 4 0.6%	845 99.2% 647 99.4%	0.442^*^
**Companion** Yes No	6 0.5% 2 1.0%	1305 99.5% 190 99.0%	0.272^*^	8 0.6% 3 1.6%	1303 99.4% 189 98.4%	0.156^*^
**Skin-to-skin contact** Yes No	2 0.2% 6 1.0%	904 99.8% 591 99.0%	0.048^*^	3 0.3% 8 1.3%	903 99.7% 589 98.7%	0.028^*^
**Timely cord clamping** Yes No	1 0.3% 7 0.6%	322 99.7% 1172 99.4%	0.460^*^	2 0.6% 9 0.8%	321 99.4% 1170 99.2%	0.567^*^

n - absolute frequency; % - relative frequency; #Peter’s chi-square test;

* Fisher’s exact test.

There was no association between Apgar and the use of good obstetric practices for women who had vaginal delivery as their mode of birth (Table 3).

**Table 3:** Analysis of the relationship between good obstetric practices associated with vaginal delivery and the Apgar score at 1 and 5 minutes, Paraná, 2021-2022

	1^st^-minute Apgar		*p* value	5^th^-minute Apgar		*p* value
	n ≤ 6 (%)	n ≥7 (%)		n ≤ 6 (%)	n ≥7 (%)	
Prenatal Yes No	3 0.4% 1 -	709 99.6% 8 100.0%	0.967^*^	5 0.7% 0 -	707 99.3% 8 100.0%	0.946^*^
**Reference hospital** Yes No	2 0.3% 1 1.4%	646 99.7% 70 98.6%	0.268^*^	4 0.6% 1 1.4%	644 99.4% 70 98.6%	0.406^*^
**Risk stratification** Low Intermediate High No record	1 0.3% 0 - 1 1.2% 1 0.5%	391 99.7% 51 100.0% 85 98.8% 190 99.5%	0.644#	2 0.5% 1 2.0% 1 1.2% 1 0.5%	390 99.5% 50 98.0% 85 98.8% 190 99.5%	0.630#
**Membrane rupture** Yes No	3 0.5% 0 -	594 99.5% 123 100.0%	0.570^*^	5 0.8% 0 -	592 99.2% 123 100.0%	0.391^*^
**Non-pharmacological method** Yes No	3 0.5% 0 -	549 99.5% 168 100%	0.450^*^	5 0.9% 0 -	547 99.1% 168 100.0%	0.264^*^
**Companion** Yes No	3 0.5% 0 -	639 99.5% 78 100.0%	0.709^*^	5 0.8% 0 -	637 99.2% 78 100.0%	0.563^*^
**Skin-to-skin contact** Yes No	2 0.4% 1 0.5%	520 99.6% 197 99.5%	0.620^*^	3 0.6% 2 1.0%	519 99.4% 196 99.0%	0.420^*^
**Timely cord clamping** Yes No	0 - 3 0.6%	242 100% 475 99.4%	0.292^*^	0 - 5 1.0%	242 100.0% 472 99.0%	0.128^*^

n - absolute frequency; % - relative frequency; #Peter’s chi-square test;

* Fisher’s exact test.

In relation to Apgar and the use of good obstetric practices used in cesarean section as a childbirth route, it was observed that 1^st^-minute and 5^th^-minute Apgar was associated with pregnant women’s connection to the reference hospital (p=0.037 and p=0.053) and skin-to-skin contact (p=0.034 and p=0.017). Thus, 5^th^-minute Apgar was associated with the presence of a companion (p=0.043) (Table 4).

**Table 4:** Analysis of the association between good obstetric practices used in cesarean section and the Apgar outcome at 1 and 5 minutes, Paraná, 2021-2022

	1^st^-minute Apgar		*p* value	5^th^-minute Apgar		*p* value
	n ≤ 6 (%)	n ≥7 (%)		n ≤ 6 (%)	n ≥7 (%)	
Prenatal Yes No	5 0.7% 0 -	763 99.3% 15 100.0%	0.908^*^	6 0.8% 0 -	762 99.2% 15 100.0%	0.890^*^
**Reference hospital** Yes No	3 0.4% 2 3.9%	728 99.6% 49 96.1%	0.037^*^	4 0.5% 2 3.9%	727 99.5% 49 96.1%	0.053^*^
**Risk stratification** Low Intermediate High No record	3 1.1% 0 0.0% 2 0.8% 0 -	275 98.9% 75 100.0% 234 99.2% 194 100.0%	0.433#	3 1.1% 0 - 3 1.3% 0 -	275 98.9% 75 100.0% 233 98.7% 194 100.0%	0.358#
**Membrane rupture** Yes No	3 0.6% 2 0.8%	526 99.4% 252 99.2%	0.522^*^	4 0.8% 2 0.8%	525 99.2% 252 99.2%	0.632^*^
**Non-pharmacological method** Yes No	2 0.7% 3 0.6%	298 99.3% 480 99.4%	0.634^*^	2 0.7% 4 0.8%	298 99.3% 479 99.2%	0.579^*^
**Companion** Yes No	3 0.4% 2 1.8%	666 99.6% 112 98.2%	0.156^*^	3 0.4% 3 2.6%	666 99.6% 111 97.4%	0.043^*^
**Skin-to-skin contact** Yes No	0 - 5 1.3%	384 100% 394 98.7%	0.034^*^	0 - 6 1.5%	384 100.0% 393 98.5%	0.017^*^
**Timely cord clamping** Yes No	1 1.2% 4 0.6%	80 98.8% 697 99.4%	0.422^*^	2 2.5% 4 0.6%	79 97.5% 697 99.4%	0.121^*^

n - absolute frequency; % - relative frequency; #Peter’s chi-square test;

* Fisher’s exact test.

## DISCUSSION

The Apgar score at the 1^st^, 5^th^, or both minutes of life of newborns assessed in this study showed a positive association with variables such as referral hospital, companion, and skin-to-skin contact, reinforcing the importance of good obstetric practices even in adverse situations. In the state of Paraná, a guide was released with recommendations for good obstetric and neonatal practices during the pandemic. Therefore, it is possible that the services studied here partially followed the recommendations^(18)^.

Apgar score assessment remains the best tool for quickly assessing newborns in the delivery room. A low score, regardless of gestational age, increases the risk of neonatal mortality^(19)^. The Apgar score is rarely studied during the pandemic, but an increase in maternal and neonatal mortality rates has been observed during this period. This increase may be related to changes in care, particularly in lowand middle-income countries^(3)^.

Prenatal care is essential for maternal and fetal well-being, preventing complications during labor and birth. It is the pregnant woman’s first contact with healthcare services, a time for building a bond with the team and accessing the entire Healthcare Network, contributing to continuous monitoring of pregnancy. Stratifying gestational risk into low-risk, intermediate-risk, and high-risk is essential to guide appropriate referrals, ensuring individualized care tailored to the complexity of each case^(20-22)^. However, prenatal care during the pandemic was negatively affected worldwide, with appointments suspended, postponed, and care locations relocated^(23)^.

In a city in southern Brazil, there was a decrease in pregnant women with adequate prenatal care (six or more consultations; basic tests in the last trimester of pregnancy; first prenatal consultation before the 16^th^ week of pregnancy), when compared to data prior to the pandemic^(24)^. Most of the women in this study attended prenatal care, despite the variations caused by the pandemic. However, there was no correlation with Apgar scores. A study conducted in Ethiopia shows that, during the pandemic, failure to attend prenatal care resulted in neonatal near miss, and 67.4% of these cases had an Apgar score below 7 at 5 minutes of life^(25)^. Despite the differences between Ethiopia and Brazil, access to healthcare services and quality of care can be common challenges.

During prenatal care, pregnant women should be advised about the referral hospital, as this helps reduce adverse outcomes^(17,26,27)^. The connection of pregnant women to the place where they will give birth is a good practice guided by the WHO, and in Brazil, it is a right guaranteed by Law 11,634/2007^(27)^. This study highlights the positive relationship between an adequate Apgar score and referral to healthcare services. Even amid the pandemic, and with the reorganization of healthcare services, the state of Paraná successfully managed to refer these women.

Leal *et al*. (2020), in an excerpt from the national survey called “*Nascer no Brasil*” (Born in Brazil), conducted before the COVID-19 pandemic, identified that the peregrination of pregnant women was associated with negative neonatal outcomes, such as neonatal near miss, prematurity, low birth weight, and Apgar <8 at the 5^th^ minute of life, with an Odds Ratio of 2.19. In addition to the peregrination faced by these women, many were denied the right to a companion^(18)^. A study carried out in 24 Brazilian maternity hospitals from March to July 2020 identified the prohibition of companions in 83.3% of hospitals, evidencing a decline in good practices^(28)^. The right to the presence of a companion during prenatal, childbirth and postpartum care is guaranteed by Law 11,108/2005 and recommended by the WHO, promoting emotional support, comfort and favoring positive maternal and neonatal outcomes^(29,30)^. This ban, although part of the health measures, accentuated barriers related to social inequities^(31)^.

This practice was also a setback internationally, as 62% of women in the European Union were restricted from having a companion, with Serbia accounting for 99.0% of cases. A study conducted in China during the COVID-19 pandemic found that women without a companion during labor were more likely to have babies with an Apgar score <7 at 1 minute^(32)^. In this study, there was no relationship between the presence of a companion and Apgar scores in vaginal deliveries. However, the presence of a companion of choice for women who had a cesarean section was a protective factor against low Apgar scores at 5 minutes. However, it is worth noting that, in this study, a companion was present in over 80% of cases in both births. Although far from ideal, the state of Paraná worked to minimize harm in the face of the pandemic’s impact, and it seems reasonable to say that, even in times of health crisis, the presence of this social figure is essential for positive neonatal outcomes.

Another good obstetric practice that contributes to positive outcomes for both women and newborns is the use of non-pharmacological pain relief methods. These non-invasive techniques, such as heat therapy, auriculotherapy, massage, and ball use, address psycho-emotional and spiritual aspects, promoting humanized childbirth, comfort, stress reduction, well-being, and autonomy, all of which contribute to increased vaginal delivery rates^(33,34)^.

In this study, there was no relationship between the Apgar score and the use of non-pharmacological methods, and although many women received these methods, this percentage still needs to be improved. The high number of women who delivered vaginally, possibly reflecting the scenario of elective cesarean sections, is treated as a probability, as it was not possible to confirm whether the cesarean section occurred before labor. The methods were not analyzed separately, which limits understanding of their effectiveness and appropriateness.

Although recommendations during the pandemic indicated that elective cesarean section should only occur in cases of dyad complications, a study carried out with 221 Brazilian women revealed that 5% of them reported the COVID-19 pandemic as a justification for indicating cesarean section, possibly due to concerns about transmission^(8,35)^. It is important to emphasize that cesarean sections without medical indications and without scientific evidence influence negative outcomes and may be associated with increased hospital stay^(36)^.

In addition to non-pharmacological methods for pain relief, immediate care for newborns in the first hours of life should be encouraged. These good obstetric care practices, such as skin-to-skin contact, encouraging breastfeeding, and timely cord clamping, are essential for reducing neonatal deaths, as fetal deaths occur in between 25 and 45% of newborns during this period. However, at the beginning of the pandemic, some guidelines on this care were controversial due to concerns about the transmission of COVID-19 to newborns^(37)^. At the height of the pandemic in Brazil, 79.1% of hospitals did not recommend skin-to-skin contact, nor breastfeeding in the first hour (87.5%)^(28)^. As these discoveries progressed, the WHO and the Ministry of Health began to reinforce the use of these practices, even in mothers infected with COVID-19, as the benefits outweigh any risks of transmission, which are, in fact, considered minimal^(37,38)^. However, we found that these practices were rarely used in the maternity hospitals studied. Skin-to-skin contact after cesarean section was significantly less frequent than skin-to-skin contact after vaginal delivery. Perhaps adaptations to hospital infrastructure during the pandemic, as well as staff reductions during this period, may have influenced this discrepancy between childbirth methods.

Skin-to-skin contact is one of the practices recommended in the ten stages for breastfeeding, consisting of the technique in which the newborn must be placed on the mother’s chest or abdomen, both without clothes or sheets that block contact^(38)^. This practice favors a good neonatal Apgar score, and even in pandemic times, it was possible to observe Apgar ≥7 when this practice was applied^(39)^, corroborating our findings.

Regarding timely umbilical cord clamping, the frequency of this practice in this study was very low, which reinforces the importance of adopting this measure. The data are similar to those found in a study in northwestern Paraná during the pandemic, as they also presented difficulties in implementing this practice, and only 8.4% of cases were performed in a timely manner^(9)^. We cannot say that this low frequency was specifically due to the COVID-19 pandemic, but these data lead us to reflect on the need to encourage the practice in services throughout the state.

### Study limitations

Data collection, conducted by different interviewers in several HR, resulted in incomplete or inconsistent records that generated sample loss, but it is emphasized that there was no compromise in the analysis.

### Contributions to perinatal care

This study contributes to the reflection on the importance of good obstetric practices and humanized care, even during pandemic events, and can also collaborate with the construction of new work processes and, thus, minimize negative outcomes for newborns.

## CONCLUSIONS

The study showed that, even in the face of the challenges posed by the COVID-19 pandemic, some good obstetric and neonatal practices were maintained, leading to positive outcomes and reinforcing the importance of evidence-based care. However, weaknesses persist in the full implementation of all practices.

Finally, the results obtained reiterate the importance of strategic actions and future studies that broaden the understanding of the effects of health emergencies, supporting effective responses from healthcare services.

## Data Availability

The research data are not available.
