# Supplementary material for: Perinatal care during the COVID-19 pandemic in the state of Paraná
Source: Rev Bras Enferm. 2026 Mar 30;79:e20250205. doi: 10.1590/0034-7167-2025-0205 (PMC13053013; doi:10.1590/0034-7167-2025-0205)
Supplement: Translation

# Translation: Assistência perinatal durante a pandemia de COVID-19 no estado do Paraná

**DOI:** 10.1590/0034-7167-2025-0205pt

**Authors:** Emily Marques Alves, Adriana Zilly, Lhays Emilly da Silva Moraes Ribeiro, Keli Regiane Tomeleri da Fonseca Pinto, Catia Campaner Ferrari Bernardy

**Affiliations:** I Universidade Estadual de Londrina. Londrina, Paraná, Brasil; II Universidade Estadual do Oeste do Paraná. Foz do Iguaçu, Paraná, Brasil

**Keywords:** Assistência Perinatal, Índice de Apgar, COVID-19, Gravidez, Cuidado Centrado no Paciente.

## Abstract

**Objetivos:**

avaliar a associação entre boas práticas obstétricas no período perinatal e a vitalidade do recém-nascido mensurada pelo Apgar durante a pandemia de COVID-19 no Paraná.

**Métodos:**

estudo transversal, recorte da pesquisa “Enfrentamento da COVID-19 e a assistência materno-infantil”, com puérperas atendidas em hospitais de cinco Regionais de Saúde do Paraná durante a pandemia de COVID-19 (2021 e 2022). A análise estatística utilizou frequências, média e desvio padrão. Associação entre Apgar e boas práticas obstétricas foi analisada pelos testes qui-quadrado e exato de Fisher.

**Resultados:**

incluíram-se 1.503 mulheres. As variáveis hospital de referência, contato pele a pele, e presença do acompanhante tiveram desfecho positivo sobre o valor do Apgar.

**Conclusões:**

apesar das adversidades da pandemia, algumas boas práticas foram mantidas, e o desfecho do Apgar foi positivo, mas destaca-se a necessidade contínua de articulação entre gestores e profissionais, e políticas públicas para garantir a qualidade do cuidado perinatal no estado.

## INTRODUÇÃO

O processo de gestar e parir é fisiológico, familiar e social, porém, com a modernização e industrialização, o modelo de cuidado proposto se tornou excessivamente medicalizado. O movimento de resgate da atenção humanizada vem sendo tema de discussão tanto no cenário mundial quanto no cenário nacional, mas ainda enfrenta desafios, e um deles foi a pandemia de COVID-19. As mudanças na assistência em saúde e as restrições no convívio social durante a pandemia intensificaram sentimentos negativos nas gestantes, elevando o risco de instabilidade emocional e, consequentemente, as complicações materno-fetais^(1,2)^.

Em 2021, a Organização Mundial da Saúde (OMS) analisou 135 países e identificou que 54% alteraram o atendimento de pré-natal e 31% modificaram os serviços de parto, contribuindo para o aumento de natimortos e mortes maternas^(3,4)^. A pandemia evidenciou ainda mais a fragilidade da assistência obstétrica global, enfraquecendo o vínculo entre as mulheres e os serviços de saúde^(5)^.

A OMS, há mais de duas décadas, recomenda diretrizes para cuidados saudáveis ao binômio. Em 2018, reforçou e atualizou as boas práticas ao parto e nascimento, por meio do manual *Intrapartum care for a positive childbirth experience*. Pautado nos direitos humanos, o manual propõe um modelo de assistência intraparto centrado nas necessidades da mulher e do recém-nascido, objetivando reduzir taxas de morbimortalidade materna e neonatal, e fortalecendo, assim, experiências seguras e positivas para mulher e sua família^(6)^.

No cenário pandêmico, houve retrocesso nas recomendações feitas pela OMS nas cinco etapas do cuidado intraparto, assim como no atendimento pré-natal. As incertezas quanto à COVID-19 levaram os serviços de saúde a adotar restrições e intervenções sem respaldo científico^(7)^. Estudos qualitativos realizados em diversas regiões do Brasil apontam que algumas medidas como a restrição dos acompanhantes durante o trabalho de parto, parto e pós-parto, critérios para o contato pele a pele, pinçamento precoce do cordão umbilical, início do teleatendimento, cancelamento ou adiamento de consultas e exames, e a não vinculação da mulher a maternidade de referência foram vistas como necessárias para manter a segurança das pacientes e dos profissionais^(7-10)^.

Na Turquia, durante a pandemia de COVID-19, as gestantes buscaram atendimento em pronto-socorro obstétrico com mais frequência devido à interrupção do pré-natal. Como consequência, tiveram aumento nos casos de bebês pequenos para a idade gestacional, Apgar < 7 no 5º minuto e encefalopatias hipóxico-isquêmicas^(11)^. Em uma metanálise, o aumento da prematuridade foi relatado como evento associado à pandemia^(12)^.

O binômio mãe e filho representa uma população ímpar nos cuidados em saúde, então é essencial reforçar a utilização das boas práticas obstétricas conquistadas ao longo dos anos^(13)^. Compreender o quanto as adaptações realizadas na assistência obstétrica durante a pandemia de COVID-19 impactaram o desfecho fetal constitui importante fonte de informação para organização de futuros processos de trabalho, além de colaborar com o Objetivo 3 (Saúde de Qualidade) dos Objetivos de Desenvolvimento Sustentável.

Assim, diante de todo o exposto e da relevância temática, questiona-se: as boas práticas na assistência às gestantes foram aplicadas durante a pandemia de COVID-19 no estado do Paraná? Como a assistência prestada à gestante neste período impactou a vitalidade fetal?

## OBJETIVOS

Avaliar a associação entre boas práticas obstétricas no período perinatal e a vitalidade do recém-nascido mensurada pelo Apgar durante a pandemia de COVID-19 no Paraná.

## MÉTODOS

### Aspectos éticos

O estudo seguiu os preceitos éticos nacionais e internacionais, sendo aprovado pelo Comitê de Ética em Pesquisa de todas as universidades envolvidas. Todas as participantes foram instruídas sobre a pesquisa, e as entrevistas só iniciaram após assinatura do Termo de Consentimento Livre e Esclarecido.

### Desenho, período e local do estudo

Trata-se de estudo transversal, recorte da pesquisa multicêntrica intitulada “Enfrentamento da COVID-19 e a assistência materno-infantil”, que analisou a assistência perinatal e o seguimento da criança durante o período da pandemia de COVID-19 entre os anos de 2021 e 2022, realizada em maternidades de baixo e alto risco de municípios pertencentes a cinco Regionais de Saúde (RS) do estado do Paraná: 3ª RS, situada ao leste; 9ª e 10ª RS, situada ao oeste; 15ª RS, situada ao noroeste; e 17ª RS, situada ao norte do estado. O método foi elaborado seguindo todas as recomendações do *STrengthening the Reporting of OBservational studies in Epidemiology*.

### População ou amostra; critérios de inclusão e exclusão

O cálculo amostral se baseou no número de partos em 2020 de cada RS, sendo considerada a margem de erro de 5%, com nível de confiança de 95% e acréscimo de margem de segurança de 10%, devido à característica da pesquisa original, por ser um estudo de seguimento e que poderia ocorrer perdas durante o período, resultando no cálculo de 2.112 mulheres.

O presente estudo utilizou dados apenas da primeira fase da pesquisa original, com entrevista presencial nas maternidades de 1.830 mulheres, sendo 432 da 3ª RS, 408, da 9ª RS, 418, da 10ª RS, 255, da 15ª RS, e 317, da 17ª RS. Utilizou-se como critério de inclusão na pesquisa multicêntrica puérperas que pariram no período da pandemia de COVID-19, residindo nos municípios das RS já citadas. Não foram incluídas mulheres que apresentavam algum tipo de agravo que interferisse no andamento da pesquisa. Para análise deste estudo, excluíram-se as participantes que tinham duas ou mais variáveis sem registro no banco de dados. A fim de avaliar a suficiência amostral após perdas, foi realizada a análise de poder *pós-hoc* para o teste qui-quadrado (χ^2^) utilizando o *software* G*Power (versão 3.1), considerando o teste qui-quadrado com 5 graus de liberdade, nível de significância de 5% (α = 0,05) e determinando o tamanho de efeito considerado grande (w = 0,5), conforme critérios de Cohen. O resultado indicou o poder estatístico de 100% (1 - β = 1,0), demonstrando que a amostra efetiva foi adequada para detectar associações com elevada magnitude entre as variáveis analisadas^(14)^.

### Protocolo do estudo

O instrumento original para coleta dos dados era composto por 112 questões; dessas, foram analisadas 20 questões. As variáveis independentes foram relacionadas a dados sociodemográficos como idade, cor da pele autorreferida, situação conjugal, grau de instrução, atividade remunerada, renda familiar e recebimento de auxílio emergencial. Outras variáveis independentes compuseram o conjunto de variáveis definidas como boas práticas obstétricas, como realização do pré-natal (mais de seis consultas e idade gestacional menor de 14 semanas na primeira consulta), encaminhamento ao hospital de referência, classificação de risco, ruptura espontânea da membrana, método não farmacológico para alívio da dor, presença de acompanhante, contato pele a pele, e clampeamento tardio do cordão umbilical. A variável desfecho foi o Apgar no 1º e 5º minuto.

O Apgar é uma medida de avaliação imediata da condição neonatal, sendo utilizada como indicativo de mortalidade, além de ser efetiva para comparar resultados de práticas obstétricas. O teste é formado por cinco variáveis: frequência cardíaca; esforço respiratório; tônus muscular; resposta reflexa; e coloração da pele. Essas variáveis pontuam entre 0 e 2 cada uma, sendo avaliadas, inicialmente, no 1º e no 5º minuto de vida do recém-nascido^(15,16)^.

Pontuações de Apgar a partir de 7 são consideradas tranquilizadoras, e representam a boa vitalidade fetal. Abaixo deste valor, é um sinal de alerta, pois está relacionado ao maior risco de asfixia perinatal e aumento de paralisia cerebral, levando ao aumento da mortalidade do recém-nascido na sua primeira semana de vida^(15-17)^. Diante disso, apresenta-se o Apgar nesta pesquisa, dicotomizado em duas categorias: ≤ 6 e ≥7 pontos.

### Análise dos resultados e estatística

Os dados foram analisados por meio de estatística descritiva e inferencial, com o auxílio do *software* IBM SPSS *Statistics*, versão 29.0. As variáveis categóricas foram apresentadas em frequências absolutas (n) e relativas (%). Já as variáveis quantitativas, tanto discretas quanto contínuas, foram descritas por meio da média e desvio padrão (DP).

Inicialmente, a análise foi conduzida considerando o total de mulheres da amostra, seguida de uma estratificação por via de nascimento. Para analisar a hipótese de associação entre a vitalidade do recém-nascido mensurada pelo Apgar e as variáveis independentes de boas práticas obstétricas, aplicou-se o teste qui-quadrado de Pearson. Quando as frequências esperadas na tabela de contingência foram inferiores a 5, utilizou-se o teste exato de Fisher.

## RESULTADOS

Foram elegíveis 1.503 participantes, excluindo-se 327 devido à ausência de dados para análise. A cor da pele predominante autodeclarada foi a branca (863; 57,4%), seguida da parda (479; 32,0%). A maior parte delas (1.315; 87,5%) possuía companheiro e já havia vivenciado de uma a duas gestações anteriores (733; 48,8%). Em relação à escolaridade, apenas 509 mulheres (33,9%) possuíam mais de 13 anos de estudo. A média de idade foi 26,61 anos (DP ± 6,01), sendo a mais jovem com 13 anos, e a mais velha, 45 anos. Em relação à ocupação, 767 mulheres (51%) não desenvolviam atividade remunerada, e a média de renda familiar foi de um a dois salários para 1.006 mulheres (66,9%) (salário mínimo vigente em 2021: R$ 1.100,00). Grande parte delas (1.131; 75,2 %) não recebia o auxílio emergencial.

O pré-natal foi frequentado por 1.480 mulheres (98,5%), sendo a maior parte (1.379; 91,7%) vinculada ao hospital de referência. As mulheres passaram pela classificação de risco na chegada ao hospital, sendo 89,94% classificadas como baixo risco. Em relação à idade gestacional no momento do nascimento, 1.368 (91,01%) estavam com mais de 37 semanas de gestação. Quanto à ruptura das membranas, identificou-se que a maior parte foi espontânea, com prevalência no parto vaginal (Tabela 1).

**Tabela 1:** Variáveis assistenciais e obstétricas de puérperas que pariram em maternidades das Regionais de Saúde do Paraná no período da pandemia de COVID-19, Paraná, 2021-2022

Variáveis	Parto vaginal (n e %)	Cesariana (n e %)
**Realização de pré-natal** Sim Não	71298,88 81,12	76898,08 151,92
**Hospital de referência** Sim Não Sem registro	64890,0 719,86 10,14	73193,35 516,51 10,14
**Classificação de risco** Habitual Intermediário Alto Sem registro	39254,44 517,08 8611,94 19126,54	27835,50 759,57 23636,14 19418,79
**Idade gestacional** Prematuro moderado (31-35 segundos) Prematuro limítrofe (36 segundos) Termo (≥37) Sem registro	50,69 141,96 64990,13 527,22	141,78 121,53 71991,82 384,87
**Ruptura espontânea das membranas** Sim Não	59782,9 12317,1	52967,6 25432,4
**Método não farmacológico** Sim Não	55276,7 16823,3	30038,3 48361,7
**Tipo método não farmacológico** Banho relaxante Massagem Uso da bola Outros métodos	48767,6 27137,6 30642,5 8912,5	14318,3 7910,1 8611,0 141,8
**Presença de acompanhante** Sim Não	64289,2 7810,8	66985,4 11414,6
**Contato pele a pele** Sim Não	52272,5 19827,5	38449,0 39951,0
**Clampeamento oportuno do cordão** Sim Não	24233,6 47866,4	8110,3 70289,7

O uso dos métodos não farmacológicos para alívio da dor foi mais frequente em mulheres que evoluíram para o parto vaginal. O banho relaxante foi a prática mais utilizada em ambos os desfechos (67,6% e 18,3% respectivamente). O acompanhante esteve presente em 1.311 dos casos (87,2%). O contato pele a pele imediatamente após o parto foi promovido para 906 mulheres (60,3%), sendo mais frequente no parto vaginal. O clampeamento oportuno do cordão esteve presente apenas em 323 dos casos (21,5%), o que evidencia o pouco uso da prática (Tabela 1). A cesariana foi a via de nascimento mais frequente (783; 52,1%).

Ao analisar a associação entre o Apgar e todas as variáveis nomeadas como boas práticas obstétricas em análise geral, consideraram-se associadas o Apgar no 1º minuto com o encaminhamento para hospital de referência (p=0,022) e o Apgar no 1º e 5º minuto com o contato pele a pele (p= 0,048 e p=0,028) (Tabela 2).

**Tabela 2:** Relação entre as boas práticas obstétricas e o Apgar no 1º e 5º minuto de vida do recém-nascido (análise geral), Paraná, 2021-2022

	Apgar 1º minuto		Valor de *p*	Apgar 5º minuto		Valor de *p*
	n ≤ 6 (%)	n ≥7 (%)		n ≤ 6 (%)	n ≥7 (%)	
Pré-natal Sim Não	8 0,5% 0 -	1472 99,5% 23 100,0%	0,884^*^	11 0,7% 0 -	1469 99,3% 2 100,0%	0,843^*^
**Hospital de referência** Sim Não	5 0,4% 3 2,5%	1374 99,6% 119 97,5%	0,022^*^	8 0,6% 3 2,5%	1371 99,4% 119 97,5%	0,053^*^
**Classificação de risco** Baixo Intermediário Alto Sem registro	4 0,6% 0 - 3 0,9% 1 0,3%	666 99,4% 126 100,0% 319 99,1% 384 99,7%	0,525#	5 0,7% 1 0,8% 4 1,2% 1 0,3%	665 99,3% 125 99,2% 318 98,8% 384 99,7%	0,504#
**Idade gestacional** Prematuro moderado Prematuro limítrofe Termo Sem registro	0 - 0 - 7 0,5% 1 1,1%	19 100% 26 100% 1361 95,5% 89 98,9%	0,844#	0 - 1 3,8% 9 0,7% 1 1,1%	19 100% 25 96,2% 1359 99,3% 1492 98,9%	0,273#
**Ruptura da membrana** Sim Não	6 0,5% 2 0,5%	1120 99,5% 375 99,5%	0,677^*^	9 0,8% 2 0,5%	1117 99,2% 375 99,5%	0,452^*^
**Método não farmacológico** Sim Não	5 0,6% 3 0,5%	847 99,4% 648 99,5%	0,516^*^	7 0,8% 4 0,6%	845 99,2% 647 99,4%	0,442^*^
**Acompanhante** Sim Não	6 0,5% 2 1,0%	1305 99,5% 190 99,0%	0,272^*^	8 0,6% 3 1,6%	1303 99,4% 189 98,4%	0,156^*^
**Contato pele a pele** Sim Não	2 0,2% 6 1,0%	904 99,8% 591 99,0%	0,048^*^	3 0,3% 8 1,3%	903 99,7% 589 98,7%	0,028^*^
**Clampeamento oportuno do cordão** Sim Não	1 0,3% 7 0,6%	322 99,7% 1172 99,4%	0,460^*^	2 0,6% 9 0,8%	321 99,4% 1170 99,2%	0,567^*^

n - frequência absoluta; % - frequência relativa; #teste qui-quadrado de Person;

* teste exato de Fisher.

Não houve associação entre o Apgar e a utilização das boas práticas obstétricas para as mulheres que tiveram o parto vaginal como via de nascimento (Tabela 3).

**Tabela 3:** Análise da relação entre as boas práticas obstétricas associadas ao parto vaginal e o desfecho Apgar no 1º e 5º minuto, Paraná, 2021-2022

	Apgar 1º minuto		Valor de *p*	Apgar 5º minuto		Valor de *p*
	n ≤ 6 (%)	n ≥7 (%)		n ≤ 6 (%)	n ≥7 (%)	
Pré-natal Sim Não	3 0,4% 1 -	709 99,6% 8 100,0%	0,967^*^	5 0,7% 0 -	707 99,3% 8 100,0%	0,946^*^
**Hospital de referência** Sim Não	2 0,3% 1 1,4%	646 99,7% 70 98,6%	0,268^*^	4 0,6% 1 1,4%	644 99,4% 70 98,6%	0,406^*^
**Classificação de risco** Baixo Intermediário Alto Sem registro	1 0,3% 0 - 1 1,2% 1 0,5%	391 99,7% 51 100,0% 85 98,8% 190 99,5%	0,644#	2 0,5% 1 2,0% 1 1,2% 1 0,5%	390 99,5% 50 98,0% 85 98,8% 190 99,5%	0,630#
**Ruptura da membrana** Sim Não	3 0,5% 0 -	594 99,5% 123 100,0%	0,570^*^	5 0,8% 0 -	592 99,2% 123 100,0%	0,391^*^
**Método não farmacológico** Sim Não	3 0,5% 0 -	549 99,5% 168 100%	0,450^*^	5 0,9% 0 -	547 99,1% 168 100,0%	0,264^*^
**Acompanhante** Sim Não	3 0,5% 0 -	639 99,5% 78 100,0%	0,709^*^	5 0,8% 0 -	637 99,2% 78 100,0%	0,563^*^
**Contato pele a pele** Sim Não	2 0,4% 1 0,5%	520 99,6% 197 99,5%	0,620^*^	3 0,6% 2 1,0%	519 99,4% 196 99,0%	0,420^*^
**Clampeamento oportuno do cordão** Sim Não	0 - 3 0,6%	242 100% 475 99,4%	0,292^*^	0 - 5 1,0%	242 100,0% 472 99,0%	0,128^*^

n - frequência absoluta; % - frequência relativa; #teste qui-quadrado de Person;

* teste exato de Fisher.

Já em relação ao Apgar e à utilização de boas práticas obstétricas utilizadas em cesariana como via de parto, observou-se que o Apgar no 1º e 5º minuto esteve associado à vinculação da gestante ao hospital de referência (p=0,037 e p=0,053) e ao contato pele a pele (p=0,034 e p=0,017). O Apgar no 5º minuto esteve associado à presença de acompanhante (p=0,043) (Tabela 4).

**Tabela 4:** Análise da associação entre as boas práticas obstétricas utilizadas na cesariana e o desfecho Apgar 1º e 5º minuto, Paraná, 2021-2022

	Apgar 1º minuto		Valor de *p*	Apgar 5º minuto		Valor de *p*
	n ≤ 6 (%)	n ≥7 (%)		n ≤ 6 (%)	n ≥7 (%)	
Pré-natal Sim Não	5 0,7% 0 -	763 99,3% 15 100,0%	0,908^*^	6 0,8% 0 -	762 99,2% 15 100,0%	0,890^*^
**Hospital de referência** Sim Não	3 0,4% 2 3,9%	728 99,6% 49 96,1%	0,037^*^	4 0,5% 2 3,9%	727 99,5% 49 96,1%	0,053^*^
**Classificação de risco** Baixo Intermediário Alto Sem registro	3 1,1% 0 0,0% 2 0,8% 0 -	275 98,9% 75 100,0% 234 99,2% 194 100,0%	0,433#	3 1,1% 0 - 3 1,3% 0 -	275 98,9% 75 100,0% 233 98,7% 194 100,0%	0,358#
**Ruptura da membrana** Sim Não	3 0,6% 2 0,8%	526 99,4% 252 99,2%	0,522^*^	4 0,8% 2 0,8%	525 99,2% 252 99,2%	0,632^*^
**Método não farmacológico** Sim Não	2 0,7% 3 0,6%	298 99,3% 480 99,4%	0,634^*^	2 0,7% 4 0,8%	298 99,3% 479 99,2%	0,579^*^
**Acompanhante** Sim Não	3 0,4% 2 1,8%	666 99,6% 112 98,2%	0,156^*^	3 0,4% 3 2,6%	666 99,6% 111 97,4%	0,043^*^
**Contato pele a pele** Sim Não	0 - 5 1,3%	384 100% 394 98,7%	0,034^*^	0 - 6 1,5%	384 100,0% 393 98,5%	0,017^*^
**Clampeamento oportuno do cordão** Sim Não	1 1,2% 4 0,6%	80 98,8% 697 99,4%	0,422^*^	2 2,5% 4 0,6%	79 97,5% 697 99,4%	0,121^*^

n - frequência absoluta; % - frequência relativa; #teste qui-quadrado de Person;

* teste exato de Fisher.

## DISCUSSÃO

O valor do Apgar no 1º, no 5º ou em ambos os minutos de vida do recém-nascido avaliado neste estudo apresentou associação positiva em relação às variáveis hospital de referência, acompanhante e contato pele a pele, reforçando a importância das boas práticas obstétricas mesmo em situações adversas. No estado do Paraná, foi lançado um guia com recomendações às boas práticas obstétricas e neonatais durante a pandemia. Desta forma, torna-se possível que os serviços aqui estudados seguiram parcialmente as recomendações lançadas^(18)^.

A avaliação do índice de Apgar ainda é a melhor ferramenta para avaliar o recém-nascido de forma ágil em sala de parto. A baixa pontuação, independentemente da idade gestacional, aumenta o risco de mortalidade neonatal^(19)^. O desfecho Apgar é pouco descrito como objeto de estudo durante a pandemia, mas observa-se o aumento da proporção de mortalidade materna e neonatal neste período. Esse aumento pode ter relação com as mudanças na assistência, principalmente em países de baixa e média renda^(3)^.

O pré-natal é fundamental para o bem-estar materno e fetal, prevenindo complicações durante o parto e o nascimento. É o primeiro contato da gestante com os serviços de saúde, sendo o momento de construção do vínculo com a equipe e o acesso à toda Rede de Atenção à Saúde, contribuindo para o monitoramento contínuo da gestação. A estratificação do risco gestacional em risco habitual, intermediário e alto risco é essencial para orientar os encaminhamentos adequados, garantindo cuidados individualizados, conforme a complexidade de cada caso^(20-22)^. Porém, a assistência pré-natal durante a pandemia foi afetada de forma negativa no mundo todo, com consultas suspensas, adiadas e locais de atendimento remanejados^(23)^.

Em uma cidade ao Sul do Brasil, houve diminuição de gestantes com pré-natal adequado (seis ou mais consultas; realização dos exames básicos no último trimestre da gravidez; primeira consulta de pré-natal antes da 16ª semana de gestação), quando comparado a dados anteriores à pandemia^(24)^. A maior parte das mulheres deste estudo frequentou o pré-natal, apesar das diversidades causadas pela pandemia, no entanto não houve relação com o Apgar. Estudo realizado na Etiópia demonstra que, durante a pandemia, a não realização do pré-natal resultou em *near miss* neonatal, e 67,4% desses casos apresentaram índice de Apgar inferior a 7 no 5º minuto de vida^(25)^. Apesar das diferenças entre Etiópia e Brasil, o acesso ao serviço de saúde e a qualidade da assistência podem ser desafios em comum.

Ainda no pré-natal, a gestante deve ser orientada sobre o hospital de referência, pois favorece a redução de desfechos adversos^(17,26,27)^. A vinculação da gestante ao local do seu parto é uma boa prática orientada pela OMS, e no Brasil, é direito garantido pela Lei nº 11.634/2007^(27)^. No presente estudo, chama atenção a relação positiva entre o índice de Apgar adequado e o referenciamento ao serviço de saúde. Mesmo em meio à pandemia, e com a reorganização dos serviços de saúde, o estado do Paraná obteve o encaminhamento efetivo dessas mulheres.

Leal *et al*. (2020), em recorte do inquérito nacional denominado “Nascer no Brasil”, realizado antes da pandemia de COVID-19, identificaram que a peregrinação de gestantes se associou a desfechos neonatais negativos, como *near miss* neonatal, prematuridade, baixo peso ao nascer e Apgar <8 no 5º minuto de vida, com uma razão de chances de 2,19. Além da peregrinação enfrentada por essas mulheres, muitas tiveram o direito ao acompanhante negado^(18)^. Estudo realizado em 24 maternidades brasileiras de março a julho de 2020 identificou a proibição de acompanhantes em 83,3% dos hospitais, evidenciando um declínio das boas práticas^(28)^. O direito à presença do acompanhante durante o pré-natal, parto e pós-parto é garantido pela Lei nº 11.108/2005 e recomendado pela OMS, promovendo apoio emocional, conforto e favorecendo desfechos maternos e neonatais positivos^(29,30)^. Essa proibição, embora parte das medidas sanitárias, acentuou barreiras relacionadas às iniquidades sociais^(31)^.

Essa prática também foi um retrocesso a nível internacional, pois 62% das mulheres da União Europeia tiveram a restrição do acompanhante, destacando a Sérvia com 99,0% dos casos. Estudo realizado na China durante a pandemia de COVID-19 observou que mulheres sem acompanhante durante o trabalho de parto tiveram uma chance maior de bebês com índice de Apgar < 7 no 1º minuto^(32)^. No presente estudo, não houve relação entre a presença de acompanhante e Apgar em partos vaginais, no entanto o acompanhante de escolha das mulheres que tiveram a cesariana como via de nascimento foi fator protetor para baixo Apgar no 5º minuto. Contudo, vale salientar que, no presente estudo, o acompanhante esteve presente em mais de 80% nas duas vias de nascimento. Mesmo longe do ideal, o estado do Paraná trabalhou para que danos fossem reduzidos diante do impacto da pandemia, e parece razoável dizer que, mesmo em tempos de crise sanitária, a presença desta figura social é fundamental para desfechos neonatais positivos.

Outra boa prática obstétrica que contribui para desfechos positivos para a mulher e o recém-nascido é o uso de métodos não farmacológicos para o alívio da dor. Essas técnicas não invasivas, como terapias térmicas, auriculoterapia, massagem e uso de bola, envolvem aspectos psicoemocionais e espirituais, promovendo a humanização do parto, conforto, redução do estresse, bem-estar e autonomia, favorecendo o aumento das taxas de parto vaginal^(33,34)^.

Neste estudo, não houve relação entre o desfecho Apgar e o uso de métodos não farmacológico, e apesar de muitas mulheres terem recebido esses métodos, ainda é uma porcentagem a ser melhorada. A grande oferta estava relacionada às mulheres que tiveram o parto vaginal, retratando possivelmente o cenário das cesáreas eletivas, mas essa informação é tratada como uma probabilidade, pois não foi possível confirmar se a cesariana ocorreu antes do trabalho de parto. Os métodos não foram analisados separadamente, o que limita a compreensão da eficácia e adequação de sua aplicação.

Embora as recomendações durante a pandemia indicaram que a cesariana eletiva deveria ocorrer apenas em casos de agravos do binômio, estudo realizado com 221 mulheres brasileiras revelou que 5% delas relataram a pandemia de COVID-19 como justificativa para indicação da cesariana, possivelmente pela preocupação da transmissão^(8,35)^. Importante ressaltar que cesarianas sem indicações médicas e sem embasamento em evidências científicas influenciam desfechos negativos e podem estar associadas ao aumento do tempo de internação^(36)^.

Além dos métodos não farmacológicos para alívio da dor, os cuidados imediatos ao recém-nascido nas primeiras horas de vida devem ser estimulados. Essas boas práticas de assistência obstétrica, como contato pele a pele, estímulo à amamentação e clampeamento oportuno do cordão, são essenciais para redução das mortes neonatais, pois, neste período, o óbito fetal ocorre entre 25 e 45%. Porém, no início da pandemia, algumas orientações sobre esses cuidados foram controversas, devido às dúvidas em relação à transmissão da COVID-19 ao recém-nascido^(37)^. No auge da pandemia no Brasil, 79,1% dos hospitais não recomendavam o contato pele a pele, tão pouco o aleitamento materno na primeira hora (87,5%)^(28)^. Com o avanço das descobertas, a OMS e o Ministério da Saúde passaram a reforçar o uso dessas práticas, mesmo em mães infectadas pela COVID-19, pois os benefícios superam quaisquer riscos de transmissão, que são, aliás, considerados mínimos^(37,38)^. No entanto, identificamos que essas práticas foram pouco utilizadas nas maternidades de estudo. O contato pele a pele após a cesariana teve frequência bastante inferior ao contato pele a pele realizado após o parto vaginal. Talvez as adaptações na estrutura hospitalar durante a pandemia, assim como a redução de funcionários neste período, possam ter influenciado nessa discrepância entre as vias de parto.

O contato pele a pele é uma das práticas indicadas nos dez passos para o aleitamento materno, consistindo na técnica em que o recém-nascido deve ficar sobre o tórax ou abdômen de sua mãe, ambos sem roupas ou lençóis que barrem o contato^(38)^. Essa prática favorece a boa pontuação do Apgar neonatal, e mesmo em tempos pandêmicos, foi possível observar Apgar ≥7 quando essa prática foi aplicada^(39)^, corroborando os nossos achados.

Quanto ao clampeamento oportuno do cordão umbilical, a frequência da prática no presente estudo foi muito baixa, o que reforça a importância da adoção dessa medida. Os dados se assemelham ao encontrado em estudo no noroeste do Paraná durante a pandemia, pois também apresentaram dificuldades em aplicar essa prática, e apenas 8,4% dos casos foram realizados de forma oportuna^(9)^. Não podemos afirmar que essa baixa frequência foi especificamente devido à pandemia de COVID-19, mas que esses dados nos levam a refletir sobre a necessidade de estimular a prática nos serviços de todo estado.

### Limitações do estudo

A coleta de dados, conduzida por diferentes entrevistadores em várias RS, resultou em registros incompletos ou inconsistentes que geraram perda amostral, mas reforça-se que não houve comprometimento na análise.

### Contribuições para a área da assistência perinatal

Este estudo contribui para a reflexão sobre a importância das boas práticas obstétricas e cuidado humanizado, mesmo em eventos pandêmicos, podendo também colaborar com a construção de novos processos de trabalho e, assim, minimizar os desfechos negativos ao recém-nascido.

## CONCLUSÕES

O estudo evidenciou que, mesmo diante dos desafios impostos pela pandemia de COVID-19, algumas das boas práticas obstétricas e neonatais foram mantidas, levando a desfechos positivos e reforçando a importância de uma assistência baseada em evidências científicas. Contudo, ainda persistem fragilidades na implementação integral de todas as práticas.

Por fim, os resultados obtidos reiteram a importância de ações estratégicas e estudos futuros que ampliem a compreensão dos efeitos de emergências sanitárias, subsidiando respostas efetivas dos serviços de saúde.

## Data Availability

Os dados de pesquisa não estão disponíveis.
